# Contrasting Evolutionary Trajectories Under Paternal Genome Elimination in Male and Female Citrus Mealybugs

**DOI:** 10.1111/mec.17826

**Published:** 2025-06-09

**Authors:** Andrew J. Mongue, Tamsin E. Woodman, Hollie Marshall, Arkadiy Garber, José C. Franco, John P. McCutcheon, Laura Ross

**Affiliations:** ^1^ Department of Entomology and Nematology University of Florida Gainesville Florida USA; ^2^ Institute for Ecology and Evolution, University of Edinburgh Edinburgh UK; ^3^ Department of Genetics and Genome Biology University of Leicester Leicester UK; ^4^ Biodesign Center for Mechanisms of Evolution and School of Life Sciences Arizona State University Tempe Arizona USA; ^5^ Departamento de Ciências e Engenharia de Biossistemas Instituto Superior de Agronomia, Universidade de Lisboa Lisbon Portugal; ^6^ Centro de Estudos Florestais Instituto Superior de Agronomia, Universidade de Lisboa Lisbon Portugal; ^7^ TERRA – Laboratório para a Sustentabilidade do Uso da Terra e dos Serviços dos Ecossistemas Lisbon Portugal

**Keywords:** adaptation, animal mating/breeding systems, insects, molecular evolution, population genetics – empirical

## Abstract

Most studies of sex‐biased genes explore their evolution in familiar chromosomal sex determination systems, leaving the evolution of sex differences under alternative reproductive systems unknown. Here we explore the system of paternal genome elimination employed by mealybugs (Hemiptera: Pseudococcidae) which have no sex chromosomes. Instead, all chromosomes are autosomal and inherited in two copies, but sex is determined by the ploidy of expression. Females express both parental alleles, but males reliably silence their paternally inherited chromosomes, creating genome‐wide haploid expression in males and diploid expression in females. Additionally, sons do not express alleles directly inherited from their fathers, potentially disrupting the evolution of male‐benefiting traits. To understand how these dynamics impact molecular evolution, we generated sex‐specific RNAseq, a new gene annotation, and whole‐genome population sequencing of the citrus mealybug, *Planococcus citri* . We found that genes expressed primarily in females hold more variation and evolve more quickly than those expressed in males or both sexes. Conversely, we found more apparent adaptation in genes expressed mainly in males than in those expressed in females. Put together, in this paternal genome elimination system there is slower change on the male side but, by increasing selective scrutiny, an increase in the degree of adaptation in these genes. These results expand our understanding of evolution in a non‐Mendelian genetic system and the data we generated should prove useful for future research on this pest insect.

## 1 Introduction

Foundational to the study of evolutionary genetics is understanding the forces that drive sequence change in organisms. These can be described in the simplest terms as either random change through genetic drift or non‐random change via selection; however, this simple binary belies the fact that molecular evolution is the amalgamation of many biological factors, both random and selective, acting on the organism expressing genotypes as phenotypes. Thus, a more nuanced statement of the goal of evolutionary genetics is to unpack the numerous, often conflicting pressures on gene evolution to understand which factors are the key drivers of change and under what conditions.

A prime example of this framework is the study of sex‐biased gene evolution (Meisel 2011). Males and females necessarily have phenotypic differences, so expression of male phenotypes in a female (and vice versa) is often costly; thus, these genes can face an inherent conflict: being beneficial in one sex but deleterious in the other (Cox and Calsbeek 2009; Van Doorn 2009). Constraining this conflict is the fact that, for the most part, males and females of the same species share the same genome. Consequently, males and females each hold genes for both sexes within their genome, meaning a male‐benefiting gene will be carried in the genome of a female at some point (and vice versa). The long‐term evolutionary resolution to this conflict is conditional, or sex‐biased, expression of genes; male‐benefiting genes become male‐biased in expression and female‐benefiting genes become female‐biased, or at the extreme end female‐limited (Wright et al. 2019). Although this solution better aligns gene expression with the sex that most benefits, conditional expression creates new countervailing forces and evolutionary genetic contradictions.

On the one hand, limiting expression to one sex decreases the proportion of the population in which these genes are exposed to selection at a given time. Under the framework of the nearly neutral theory of molecular evolution, this reduction should make selection less efficient and increase fixation of nonadaptive alleles through drift for sex‐biased genes compared to those expressed in both sexes (Baines et al. 2008; Dapper and Wade 2016; Ohta 1992). On the other hand, many of these biased genes encode reproductive traits and are often observed or predicted to evolve more quickly and adaptively than other genes, thanks to their roles in species boundary formation and sexual selection (Civetta and Singh 1995; Swanson and Vacquier 2002; Wright et al. 2015). Thus, understanding which factors are most important to the evolution of sex‐biased genes is challenging to say the least.

In practice, the study of sex‐biased gene evolution is usually associated with the study of sex chromosomes (Albritton et al. 2014; Ellegren 2011; Sackton et al. 2014) in part for the practical reason that these chromosomes tend to be enriched for sex‐biased genes compared to the autosomes (Allen et al. 2013; Jaquiéry et al. 2013; Mongue and Walters 2017). And indeed, there is a wealth of research exploring the role of adaptation and drift in the evolution of sex‐biased genes on the sex chromosomes (Dean et al. 2015; Mank et al. 2009; Meisel and Connallon 2013; Mongue et al. 2022; Mongue and Baird 2024; Rousselle et al. 2016; Whittle et al. 2020). Although patterns like increased rates of molecular evolution of sex‐biased genes have emerged in the study of sex chromosomes, they also come with a number of confounding biological factors that set them apart from the autosomes including smaller population sizes in any given species (Mank et al. 2010; Vicoso and Charlesworth 2009), sex‐biased recombination rates in some taxa (Danzmann et al. 2019; John et al. 2016; Turner and Sheppard 1975), and variable gene regulation compared to the autosomes (Disteche 2012; Gu and Walters 2017). More systematic study of sex chromosomes in a wider array of taxa will doubtless help understand the interplay of these facets, but to better understand what factors most impact sex‐biased genes, it would be valuable to explore their evolution under a variety of genomic architectures. In particular, we would like to introduce a new model, the citrus mealybug, to explore the evolution of sex‐biased genes in the absence of sex chromosomes.

### 1.1 The Unique Genetics of the Mealybug Model System

**FIGURE 1:**
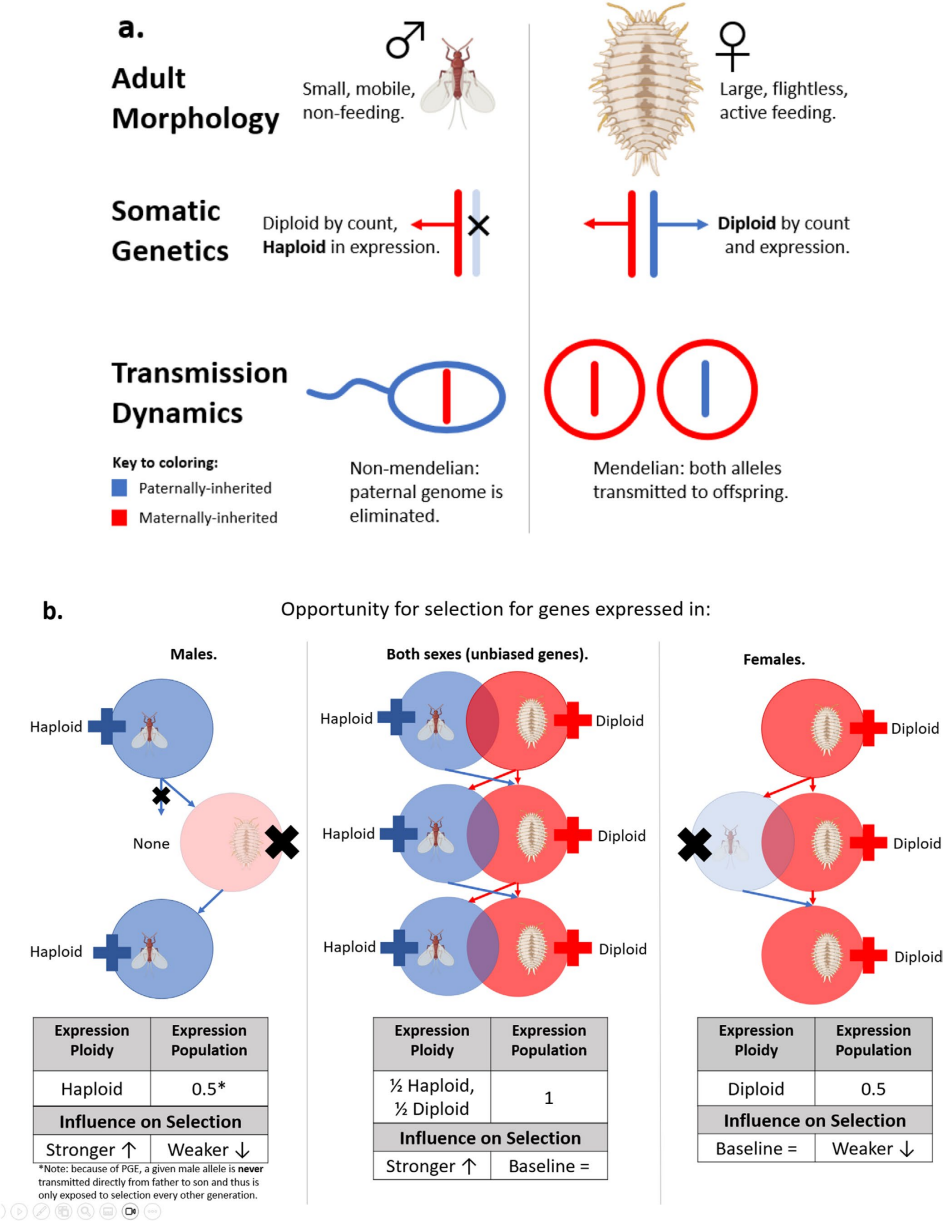
Introduction to the sex determination system of citrus mealybugs and the conditions it creates for sex‐biased genes. (a) Overview of paternal genome elimination (PGE). Males and females have extremely different morphologies despite a lack of sex chromosomes. Throughout their lives, males only express one copy of their genome, their maternal haplotype. At the time of reproduction, only maternal alleles are passed to the next generation by males. Females express and pass on both maternal and paternal alleles. (b) Considerations for selection on sex‐biased genes under PGE. Alleles are exposed to selection (i.e., expressed, + signs) under very different conditions depending on the sex of expression. Although only sex‐limited genes should be exposed strictly to one selective regimen, sex‐biased genes should face either male‐ or female‐selective conditions more often than the reserve. Specifically, male‐biased genes are expressed in roughly half the population under an equal sex ratio, are haploid in expression most of the time, and must pass from father to daughter to be expressed in a male again in the following generation. Unbiased genes are expressed in the full population, but in a haploid or diploid state depending on whether they are in males or females, respectively. Finally, female‐biased genes are also expressed in half the population and predominantly in a diploid state. These conditions should have differing impacts on the strength of selection. Predictions (stronger/weaker) are shown relative to a diploid unbiased allele baseline. Representations of mealybugs were created with biorender.com.

The group containing the citrus mealybug, the scale insects (Hemiptera: Coccoidea), likely possessed X sex chromosomes ancestrally, as other Hemiptera and early diverging scale insects still employ this sex determination mechanism (Blackmon et al. 2017; Gavrilov 2007; Nur 1980). But many scale insects, including the citrus mealybug, *Planococcus citri* , employ an unusual alternative to sex chromosomes known as the paternal genome elimination (PGE) system of sex determination (Blackmon et al. 2017; Nur 1980; Ross et al. 2022; Tree of Sex Consortium 2014). In this system, males and females share the entirety of their genome, i.e., all chromosomes are autosomal and found in both sexes but differ in the number of copies expressed in and passed on to offspring (Figure 1a). The initial sex difference is that embryos that develop as males transcriptionally silence their paternally inherited alleles (de la Filia et al. 2021; Nelson Rees 1962) and fail to pass these silenced alleles onto the next generation; females, in contrast, express and transmit both maternally and paternally inherited alleles (Herrick and Seger 1999; Hughes‐Schrader 1948; Nur 1966; Schrader 1921).

Although the precise molecular mechanisms controlling PGE and the initial evolutionary changes that enabled it are still unknown (Herbette and Ross 2023), the outcome is simple from a population genetic point of view. The mealybug PGE system is ultimately a form of haplodiploidy. However, unlike the most prominent examples of haplodiploidy found in Hymenoptera, especially among eusocial insects (Crozier et al. 1987; Gadau et al. 2000), both sexes of mealybug derive from sexual reproduction. Selective pressures are not complicated by complex social castes that create multiple distinct phenotypes for one sex, and reproduction is not restricted to a small subset of the population. Instead, all females and males are reproductively active, with extreme phenotypic dimorphism between the sexes (Figure 1a), a feature shared across scale insects (Kosztarab and Watson 1994; Mongue et al. 2021, 2024).

To a first approximation, PGE makes the entire mealybug genome similar to an X sex chromosome in some senses: it is expressed in the haploid state in males and spends more time over the generations in females than in males (Hitchcock et al. 2022). But there are significant differences that make the population genetic study of PGE an important contrast to the study of sex chromosomes. First, the sex chromosomes are predicted (Vicoso and Charlesworth 2009) and often observed (Mank et al. 2010; Mongue et al. 2022) to have a smaller effective population size than the autosomes. Second, owing to sex‐limited recombination in some lineages, the sex chromosomes often have different effective recombination rates compared to the autosomes (John et al. 2016; Turner and Sheppard 1975). Third, sex chromosomes often evolve expression regulation systems to compensate for imbalances in copy number between the autosomes and sex chromosomes in the haploid sex (Disteche 2012; Gu et al. 2019). In each of the above sex chromosome scenarios, the study of sex‐biased genes is complicated by the fact that the sex chromosomes evolve under different dynamics and typically hold proportionally more sex‐biased genes than the autosomes (Allen et al. 2013; Mongue and Baird 2024; Mongue and Walters 2017). The whole‐genome nature of PGE ensures that sex‐biased genes have the same background evolutionary dynamic regardless of linkage. For instance, recombination only occurs in female mealybugs (Bongiorni et al. 2004), but because PGE applies to the whole genome, recombination rates are not biased toward one part of the genome with more sex‐biased genes.

To explore this unique form of sex determination, we study how sex‐biased genes evolve under PGE, which creates differing and contradictory selective pressures on genes depending on their sex of expression (Figure 1b). In particular, we ask (1) do genes with sex‐biased expression generally evolve differently than those expressed in both sexes? (2) Is the evolution of sex‐biased genes consistent with predictions based on the ploidy of expression? (3) How do these two factors interact to create the overall selective dynamic of paternal genome evolution at different evolutionary timescales? While answering these questions cannot definitively implicate PGE as the key factor driving molecular evolution in mealybugs, these are important steps to understanding how selection acts in an understudied clade.

## 2 Methods

### 2.1 Study System and Sample Collection

We studied the easily cultivated (Mahmoud et al. 2017) and widely invasive citrus mealybug, *Planococcus citri* . We reared colonies to generate RNA sequencing from nymphal and adult males and females as well as proteomic data from the bacteriome and residual body tissue. This resolution of data was not sufficient for further molecular evolutionary analysis, and ultimately, the data were used for evidence in gene annotation. For the RNAseq, mealybug colonies (strain CP1‐2) were kept in a temperature and light‐controlled room at 25°C with a 16:8 light: dark photoperiod. They were fed *ad libitum* on sprouted Albert Bartlett Anya seed potatoes. Males and females were separated before sexual maturity to ensure virginity. Male and female nymphs were collected 18 days post‐egg laying. Adult males were collected daily upon eclosion (aged 24–28 days), and adult females were collected between 32 and 35 days old. RNA was extracted from 30 to 50 3rd instar males, 20 3rd instar females, 30–50 adult males, and 3 adult females per replicate, following a custom protocol (https://github.com/agdelafilia/wet_lab). We have previously shown that this does not affect downstream analysis (Bain et al. 2021). RNA quantity and quality were checked using Nanodrop and Qubit fluorometers as well as via gel electrophoresis. Samples were sent to BGI Tech Solution Co. Ltd. (Hong Kong) for library preparation and sequencing. Samples were sequenced to a depth of 50 million reads per sample on a DNBSEQ platform using 150 bp paired‐end reads. We used these above data to provide evidence for gene annotation and, in the case of the RNAseq, establish the sex bias in gene expression, as described below.

To understand the molecular evolution in nature, we sampled wild‐caught individuals. We collected and sequenced one adult female per tree across a transect of a single citrus grove in Portugal. We extracted DNA from whole body tissues via a simple salt and alcohol precipitation and sequenced on an Illumina Novaseq to roughly 20× depth with Novogene (Cambridge, UK). See data availability for accession numbers.

We worked with the Darwin Tree of Life initiative to generate a high‐quality genome assembly from a lab‐reared colony of *P. citri* (Ross et al. 2024). In brief, they used a mixture of PacBio HiFi long reads and Hi‐C linked Illumina reads to generate an assembly that contains 5 chromosomal scaffolds. This external group did not do in‐depth gene annotation, so we annotated the final genome with the BRAKER pipeline (Brůna et al. 2021), using as evidence protein sequences from the related mealybug *Phenacoccus solenopsis* (Li et al. 2020), proteins from a previous annotation of *P. citri* that were supported by mass spectrometry data, and newly generated RNAseq from nymphs and adults, described directly below. We assessed the completeness of the annotation with BUSCO v4.1.4 and the hemiptera_odb10 dataset of orthologs (Manni et al. 2021). We also generated functional annotations for follow‐up research using Interproscan v5.53‐87.0 (Jones et al. 2014).

### 2.2 Differential Gene Expression

We carried out differential gene expression analyses to identify sex‐biased genes. Our expression dataset contained 4 biological replicates of third instar males (the earliest stage as which sex is morphologically distinguishable), 6 third instar females, 3 adult males, and 6 adult females; the smaller size of males resulted in higher failure rates of extraction and library prep, creating uneven datasets between sexes. We aligned these expression datasets to the reference and annotation generated above and used RSEM v.1.3.3 (Li and Dewey 2011) implementing STAR v.2.7.10a (Dobin et al. 2013) to quantify gene expression. We then used the R package DESeq2 v.1.40.1 (Love et al. 2014) to identify differentially expressed genes between comparisons, using a Log_2_ fold‐change > 1.5 and an adjusted *p*‐value < 0.05. Specifically, we completed pairwise contrasts for male vs. female expression in nymphs and in adults separately. We classified genes that passed our expression cutoffs but did not show significant sex bias in expression as unbiased (i.e., expressed equally in both sexes) in subsequent analyses.

Not combining data for nymphs and adults gave us the opportunity to explore the consistency of sex‐biased gene expression across life stages. Because our expectations for molecular evolution are based on the ploidy of expression, such consistency is meaningful. For instance, a gene identified as significantly male‐biased in adults, but expressed in both sexes in nymphs, likely faces a different exposure to selection than one consistently male‐biased in both juveniles and adults. We grouped genes into the categories shown in Table 1 based on significant results in nymphs and adults respectively. In the main analyses, we focus on the subset of genes with consistent bias in juveniles and adults (bolded diagonal), because our intention is to study the effects of PGE and ploidy‐differences throughout life.

**TABLE 1:** Assignment of sex‐biased genes based on expression profiles in both nymphal and adult mealybugs.

	Adult female‐biased	Adult unbiased	Adult male‐biased
Nymph female‐biased	**Female‐biased**	Partially female‐biased	Sex reversal
Nymph unbiased	Partially female‐biased	**Unbiased**	Partially male‐biased
Nymph male‐biased	Sex reversal	Partially male‐biased	**Male‐biased**

*Note:* We completed differential expression analyses between the sexes for nymphs and adults separately, then compared results. Categories along the diagonal showed consistent expression patterns, while those off‐diagonal disagreed between nymphs and adults. We focus analyses on those with consistent expression (bold above) in the main text. Exploration of off‐diagonal categories is in the supplement.

These genes act as our proxy for the effects of selection in males or females, as a sex‐biased gene should predominantly be exposed to the selective conditions associated with that sex; however, to the extent that this assumption is violated, it should make the selective pressures more similar between sexes. Thus, significant differences observed in downstream tests of molecular evolution occur in spite of, rather than because of, any simplifying assumptions made here. Still, out of an abundance of caution, we explore the patterns of evolution of a smaller subset of strictly sex‐limited genes in the supplement to demonstrate the consistency of our findings. Finally, for completeness, we also explore the small subset of genes that partially escape silencing in males (de la Filia et al. 2021), but finding no meaningful patterns, we report it in the Supporting Information.

### 2.3 Population genomic analysis pipeline

We received adapter‐ and quality‐trimmed reads from the sequencing company, making them alignment‐ready. We took this data through a pipeline established by Mongue et al. (2019, 2022) and Mongue and Kawahara (2022) to generate high‐quality SNV (single nucleotide variant) calls. We generated both within‐population (polymorphism) and between‐species (divergence) variant calls. For the latter, we used a previously sequenced sample (de la Filia et al. 2021) of the related mealybug, *Planococcus ficus*. The process is described in detail below.

### 2.4 Polymorphism

We aligned conspecific reads to the reference with Bowtie2 version 2.3.5.1's *very‐sensitive‐local* alignment settings (Langmead and Salzberg 2012), sorted alignments and removed optical duplicate reads with Picard tools version 2.18.7 (Wysoker et al. 2013), then took these alignments through the Genome Analysis Toolkit version 4.1.9.0's best practices pipeline for generating high‐quality SNP calls (McKenna et al. 2010). Lacking a set of “known” SNPs for *P. citri* , we implemented a hard quality filter with the following parameters: Quality by Depth > 2.0, Fisher Strand‐bias < 60, and Mapping Quality > 40. We continued analyses with SNPs that passed all of these filters.

Next, we used our newly generated gene annotations to create a custom database in the program SnpEff version 5.1 (Cingolani et al. 2012). This database parsed coding sequences into codons and classified all SNPs by their predicted amino acid effect (synonymous, missense, nonsense, etc.). To deal with cases of multiple transcripts belonging to a single gene, we used the ‐canon option to select the longest transcript as the canonical one. We took the subset of SNPs labeled “synonymous” to use as synonymous variants and considered only “missense” SNPs as nonsynonymous under the logic that frameshifts and changes to start and stop codons are likely to cause large fitness effects across a gene and violate assumptions of SNP‐based tests of selection.

Finally, some downstream tests for adaptation require information about the frequency of derived (new mutant) alleles compared to their ancestral state. Standard variant call format files (vcfs) contain estimates of *non‐reference* allele frequency (AF), i.e., those that differ from the reference sequence. In the case that the reference allele is ancestral, then this frequency is the same as the derived AF. If, however, the reference genome carries a derived allele, then AF represents the frequency of the ancestral allele instead. This is easily corrected by taking the complement (1—AF) but requires knowledge of which alleles are ancestral and which are derived. We compared our polymorphism dataset with the divergence dataset below to infer ancestral allele state via parsimony as follows. If a position in the genome had a variant in the polymorphism dataset but not the divergence data, it meant that the outgroup carried the reference allele, and we concluded that the non‐reference allele was derived. On the other hand, if the polymorphism and divergence dataset shared the position and identity of an allele (ancestral polymorphisms in the section below), we inferred that the reference allele was ancestral and corrected the allele frequency to 1—AF.

With these corrections completed, we tallied counts of nonsynonymous polymorphisms (Pn) and synonymous polymorphisms (Ps) for each gene, both overall (for all variant frequencies) and using a sliding cutoff which excluded variants with a derived allele frequency < X where X ranged from 0.1 to 0.9 in increments of 0.1, using scripts previously developed and published by Mongue and Baird (2024). We also used an R script developed and published in Mongue et al. (2019) to label the degeneracy of each coding site position and sum for each gene to give us the number of nonsynonymous and synonymous sites for each gene. With these values, we could normalize variant counts by the number of sites within a given gene. We combined these values to calculate pN/pS, or the scaled nonsynonymous polymorphism rate. This value is the conceptual equivalent of dN/dS within species and measures the rate of nonsynonymous polymorphism relative to synonymous polymorphism with each of the two categories normalized by the number of nonsynonymous or synonymous sites in a gene, respectively.

We tested for differences between the classes of genes (i.e., male‐biased, unbiased, female‐biased) using the non‐parametric equivalent of an ANOVA, the Kruskal–Wallis test, first for pN (nonsynonymous polymorphisms per nonsynonymous site), then pS (synonymous polymorphisms per synonymous site), and finally for pN/pS together. For significant results, we performed a post hoc Dunn's test using the dunnTest() function in the R package “FSA” (Ogle and Ogle 2017) and used a threshold of *p* < 0.05 on Holm‐Bonferroni adjusted *p*‐values to determine significance of pairwise differences.

### 2.5 Divergence

To obtain high‐quality divergences (substitutions between species), we used a very similar pipeline to the one for polymorphism data. The starting point sequencing came from a single female *Planococcus ficus*, previously sequenced (de la Filia et al. 2021). Thanks to the relatively recent divergence time between *P. ficus* and *P. citri* , we were able to use the much more developed resources of the latter to simplify analyses. We aligned this outgroup to the *P. citri* genome again using bowtie2 and an identical set of downstream filtering parameters to generate and annotate a set of high‐quality single nucleotide variants (in this case, divergences). We used the *P. citri* annotation to categorize these variants as synonymous or nonsynonymous, then sent them through an additional curation step to remove false divergences.

Aside from the methodological convenience, the similarity between the two species created a concern that ancestral polymorphism shared by both species could be included in naïve Dn and Ds counts. To remove these problem variants, we curated our dataset using the following logic. For the 330,360 homozygous coding sequence variants, we checked whether they shared both position (scaffold and base number) and identity (A,G,C,T) of a variant in the polymorphism dataset (e.g., REF = A, Focal.ALT = T, outgroup.ALT = T). If so, we marked these variants as ancestral to the split between species (ancestral polymorphism) and removed them from our count of divergences. If either the position or identity differed between outgroup and focal variants, we considered the divergence valid. Ultimately, 91.8% of homozygous variants passed this curation step.

For heterozygous variants, we again first parsed whether or not the outgroup variant intersected a focal variant. If not, then we considered the variant identity. Specifically, if a heterozygous outgroup variant was called as a single base (e.g., REF = A, ALT = T), this implied that the outgroup shared a reference allele (so the full genotype of the previous example would be A,T), and we removed these variants as false divergences (outgroup polymorphisms). In some cases, however, the heterozygous outgroup site had two allele calls (e.g., REF = A, ALT = T,C). These were far rarer but could represent cases of a true divergence between species followed by a polymorphism at the same site. As a practical matter, however, we had no way of assessing which variant was the divergence and which the polymorphism, so we kept only sites for which the two outgroup alleles had the same codon effect (synonymous or nonsynonymous). In that way, we could count the category of divergence without having to confidently determine to which of the two variants it belonged. In total, there were only 411 of these tri‐allelic true variants in our dataset. Finally, we considered heterozygous outgroup variants that did coincide with focal polymorphisms. In these cases, the outgroup had to contain neither the reference allele nor the focal polymorphism(s) to be considered a true divergence. In other words, the only passing variants in this category were quadrallelic sites, which are vanishingly rare. In total, we found two valid 2 variants out of our 23,770 heterozygous coding sequence variants. Combined with the preceding class of tri‐allelic passing variants, only 1.7% of heterozygous outgroup variants represented true divergences. Because homozygous variants were far more common, however, a total of 85.7% of our overall outgroup variants passed this curation step, leaving us with 303,981 coding sequence divergences for use in downstream analyses. As with the polymorphism data, we used the non‐parametric Kruskal–Wallis test and post hoc Dunn's test to determine which groups (dN, dS, and dN/dS) were different from each other on a pairwise basis.

### 2.6 Adaptation

Finally, we combined polymorphism and divergence data to estimate adaptive molecular evolution. We computed the proportion of amino acid substitutions driven by positive selection, *α*, in multiple ways. First, we used a simple per‐gene calculation (Smith and Eyre‐Walker 2002): *α* = 1 – (Pn/Ps)/(Dn/Ds). This statistic assumes that polymorphisms should represent mostly neutral variation, but in practice many populations show an excess of polymorphisms often attributed to a wealth of weakly deleterious polymorphisms that have not yet been removed from the population by selection (Charlesworth and Eyre‐Walker 2008); this dynamic can result in negative *α* values which are not strictly defined and obscure the true proportion of adaptive substitution. These weakly deleterious variants should be almost exclusively nonsynonymous, as selection should only act directly on synonymous variants in rare cases of biased codon usage (Hershberg and Petrov 2008). If different sex‐bias classes are exposed to different selective forces from codon bias, this could differentially impact *α*, most straightforwardly, causing higher estimated alpha when there is more selection on synonymous polymorphisms (Matsumoto et al. 2016). To explore the extent of potential codon bias, we compared the base pair composition of 3rd position, four‐fold degenerate sites between male‐biased and female‐biased genes (see Supporting Information). To explore the extent of weakly deleterious nonsynonymous variants in our dataset, we first plotted scaled nonsynonymous polymorphisms for each sex‐bias class (female‐biased, unbiased, and male‐biased) of genes as a function of their derived AF (Figure S1). Based on evidence of excess polymorphism at frequencies < 0.2 in all gene classes, we chose this as a cutoff and recalculated *α* = 1 – (Pn > 0.2/Ps)/(Dn/Ds). This approach is similar to the heuristic used by Charlesworth and Eyre‐Walker (2008), who employed a cutoff of Pn > 0.15 to exclude weakly deleterious polymorphisms. To be sure, this will upwardly bias the inferred *α* in relation to how many nonsynonymous polymorphisms are low frequency; however, our aim is not to give an unbiased estimate of the true proportion of substitutions driven by positive selection, but rather to use the biases in *α* to make inferences about relative differences in the selective landscapes for different gene classes. To that end, we present the results of these two calculations (simple *α* and *α* removing low‐frequency polymorphisms) in the main text. For added confidence in the robustness of the pattern, we explore a stricter cutoff, removing Pn < 0.4, in the supplement. These *α* calculations are compound statistics involving ratios and small count data, so, as with other population genetic statistics, we used non‐parametric statistics to assess significant differences using a Kruskal–Wallis test and post hoc Dunn's test if the former indicated significant differences.

### 2.7 Long‐Term Evolution: Orthology

In the above analyses, we saw significant differences in the molecular evolution of sex‐biased gene classes in the short term (polymorphisms within a *P. citri* population) and over the medium term (divergence and adaptation between two *Planococcus* species). To explore these patterns across deeper evolutionary time, we compared the evolutionary conservation of sex‐biased genes across scale insect families. There are relatively few genomic resources for scale insects, but we found a high‐quality gene annotation for the Chinese wax scale, *Ericerus pela* (Yang et al. 2019), a member of the family Coccidae, which last shared a common ancestor with *P. citri* (Pseudococcidae) > 150 mya in the Jurassic (Vea and Grimaldi 2016) but shares PGE as a sex determination system (Gavrilov 2007). We called 1‐to‐1 orthologs between the two species' gene annotations using the tool proteinortho (Lechner et al. 2011) v5.16 and then used a *X* ^2^ test of independence to determine whether different sex‐biased classes, as defined in *P. citri* , were conserved at similar or different rates.

## 3 Results

### 3.1 Gene Annotation

We annotated 21,273 genes encoding 22,918 proteins and benchmarked this annotation against the BUSCO hemiptera_odb10 dataset of conserved orthologs. We recovered C: 95.0% [S: 77.9%, D: 17.1%], F: 0.5%, M: 4.5%. We note that BUSCO duplication scores are strongly impacted by the number of alternative transcripts in an annotation, so we parsed out the longest transcript per annotated gene and then reran the BUSCO search, which lowered the apparent duplication rate to 8.8%. We do not include this trimmed annotation as a resource because it discards potentially meaningful information about alternatively spliced genes; we only used this as a means to get a more accurate sense of ortholog duplication in the genome. Indeed, the duplication rate for this annotation is only slightly higher than the 7.6% BUSCO duplication rate reported for the genome assembly itself (Ross et al. 2024). More generally, at either duplication rate, this new annotation is undoubtedly a marked improvement over the previous annotation of 40,620 genes based on a much more fragmented assembly (https://ensembl.mealybug.org/Planococcus_citri_pcitriv1/Info/Index). We also present functional annotations in the supplementary interproscan.csv file and an abbreviated list of enriched GO terms for each of the bias classes in the Supporting Information.

### 3.2 Differential Gene Expression

We assessed differential gene expression between males and females in nymphs and adults separately. Of the 21,273 genes in the annotation, we excluded 6676 genes mainly for low or no expression, but also for discrepancies in gene model lengths in a small number of cases (i.e., the exonic length was not divisible by three). This left us with 14,597 genes to analyze. Next, we carried out differential expression analyses separately for nymphal males vs. females and adult males vs. females, then cross‐referenced the results from the two life stages. There was strong agreement between life stages over sex bias of expression. Where nymphal and adult data disagreed, it typically manifested as more sex‐biased genes in adults than nymphs. Cases of reversal of sex bias (e.g., female‐biased in nymphs but male‐biased in adults) were a small fraction of expressed genes (Table 2). For the remainder of the analyses, we focused on 7322 genes with a consistent sex bias in expression: 1003 male‐biased (13.7%), 5155 unbiased (70.4%), and 1164 female‐biased genes (15.9%). Although this approach excludes many genes in the genome, it represents the set of genes for which we have the highest confidence in expression bias and gives us large samples with which to make comparisons between bias classes.

**TABLE 2:** Categorization of sex‐biased genes from differential expression analyses.

	Adult female‐biased	Adult unbiased	Adult male‐biased
Nymph female‐biased	**1164**	453	143
Nymph unbiased	2050	**5155**	2097
Nymph male‐biased	189	343	**1003**

*Note:* We performed two separate but equivalent differential expression analyses to determine whether a given gene showed significant sex bias in expression: one using nymphal RNAseq and one using adult data. Values along the diagonal showed agreement in both tests. Unbiased genes are those expressed in both sexes equally. In the main text, we focus on genes with consistent sex bias in expression in both life stages (bold). Colors follow the color of sex‐biased genes throughout figures, with lighter shades being inconsistently biased genes.

### 3.3 Short‐Term Evolution: Sex Differences in Polymorphism

Using the above definitions of sex‐biased genes, we compared polymorphism across the three consistently biased expression classes. We first examined nonsynonymous (i.e., putatively non‐neutral) variation and synonymous (i.e., putatively neutral) variation separately, before combining the variation data to test the relative rate of nonsynonymous to synonymous variation within species (pN/pS).

**FIGURE 2:**
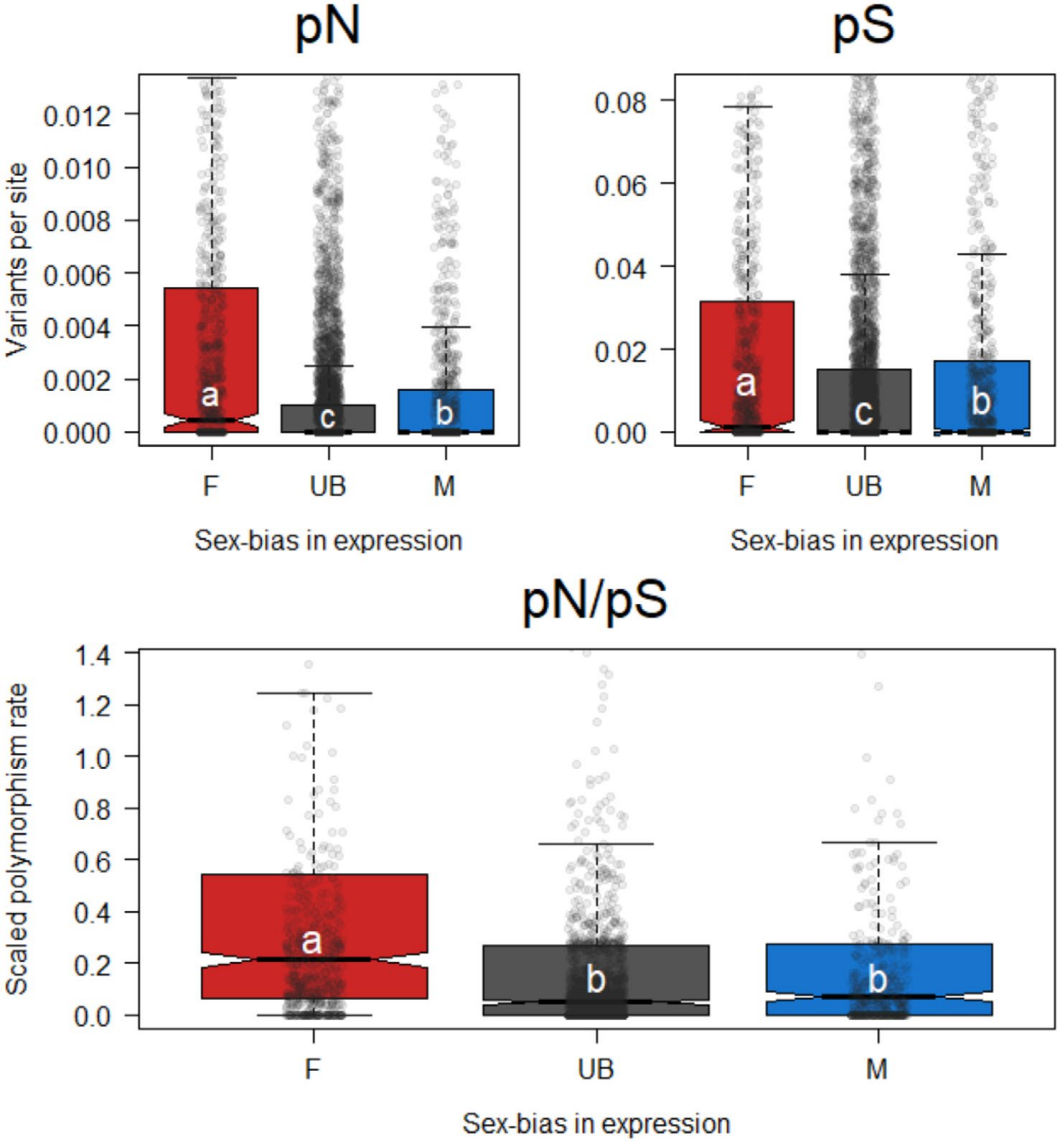
Polymorphism and sex‐biased gene expression. Colors correspond to the bias classes defined in Table 1 and letters denote significant differences such that classes with different letters are significantly different and a > b > c. Semi‐transparent points show individual gene values. Top left: Nonsynonymous variants per nonsynonymous site (pN). Female‐biased genes hold the most variation, followed by male‐biased genes, and then unbiased genes hold the least. Top right: Synonymous variants per synonymous site (pS). Synonymous variation follows the same pattern as nonsynonymous variation: Female‐biased genes hold the most, followed by male‐biased and unbiased genes. Bottom: Scaled polymorphism rate (pN/pS). Considering both classes together, female‐biased genes hold more scaled variation than either unbiased or male‐biased genes, which do not differ from each other.

For nonsynonymous variation, we found strong differences between the classes ($X_{2}^{2}$ = 234.57, *p* < 0.0001). Post hoc testing revealed the highest rates of scaled nonsynonymous variation in female‐biased genes, followed by male‐biased genes, with unbiased genes holding the least (Table 3 Top, Figure 2 Top Left). Considering synonymous polymorphism alone, the sex‐bias classes again differed from each other ($X_{2}^{2}$ = 44.74, *p* < 0.0001) in the same pattern as nonsynonymous polymorphism (Table 3 middle and Figure 2 top right). Finally, combining pN and pS into the ratio pN/pS, we found a strong overall effect of sex bias on the ratio of nonsynonymous to synonymous variation ($X_{2}^{2}$ = 148.43, *p* < 0.0001). These differences followed the differences observed in pN, with female‐biased genes holding relatively more nonsynonymous variation than unbiased or male‐biased genes (Figure 2 bottom, Table 3 Bottom).

**TABLE 3:** Holm‐Bonferroni adjusted *p*‐values for pairwise differences between sex‐bias classes for within species variation (polymorphisms).

Statistic	Female‐biased vs. unbiased	Unbiased vs. male‐biased	Female‐biased vs. male‐biased
pN	**< 0.0001**	**0.004**	**< 0.0001**
pS	**< 0.0001**	**0.021**	**0.004**
pN/pS	**< 0.0001**	0.113	**< 0.0001**

*Note:* Bolded values are significant at a *p* < 0.05 threshold. Top row: Nonsynonymous variants per nonsynonymous site (pN). Middle: Synonymous polymorphisms per synonymous site (pS). Bottom: Scaled polymorphism (pN/pS).

### 3.4 Long‐Term Evolution: Sex Differences in Divergence

**FIGURE 3:**
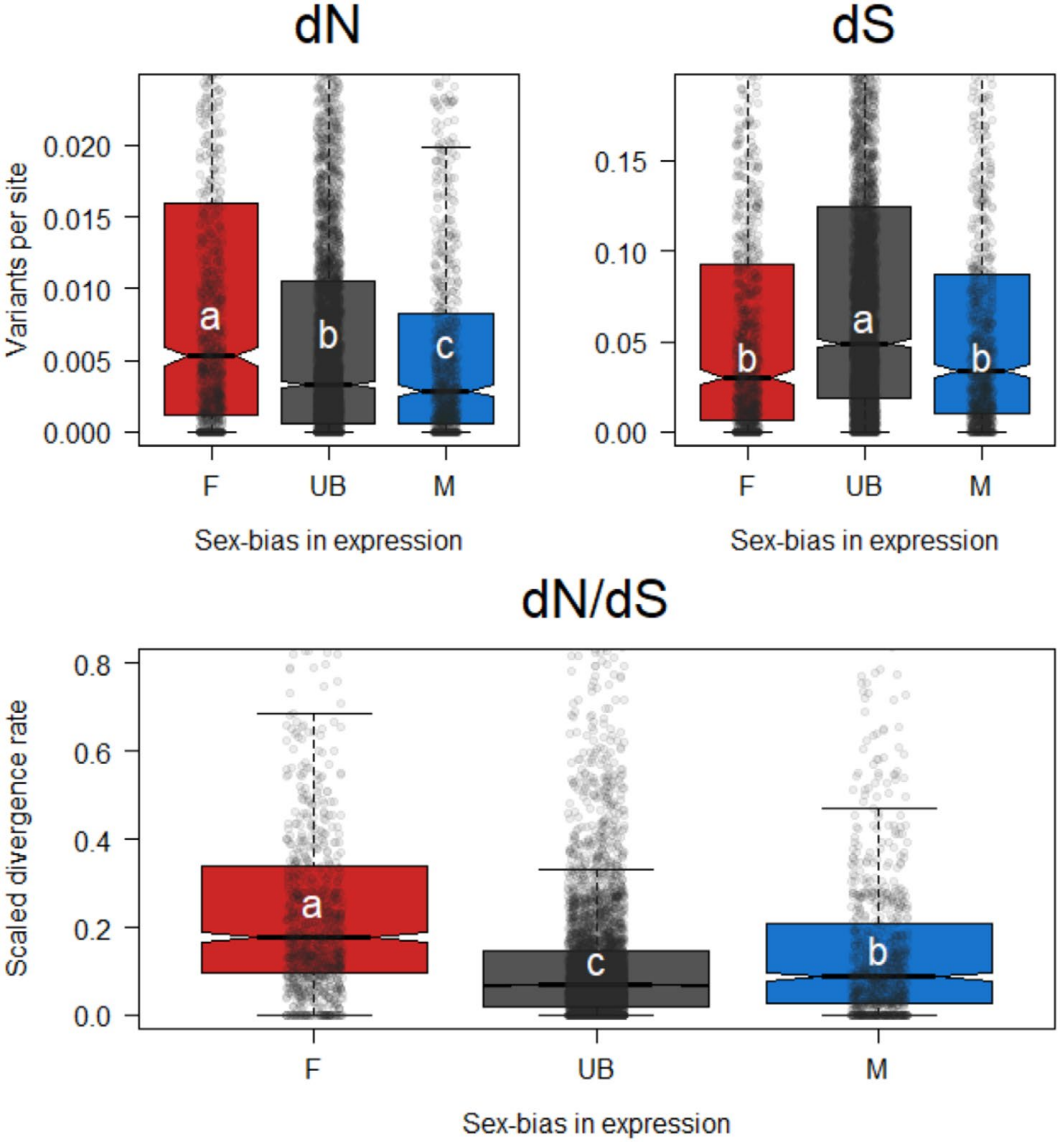
Divergence rates across sex‐bias classes. Top left: Nonsynonymous substitutions per nonsynonymous site. Female‐biased genes show the most nonsynonymous change, followed by unbiased genes, with male‐biased genes showing the least nonsynonymous change. Top right: Synonymous substitutions per synonymous site. Female‐biased and male‐biased genes show less scaled synonymous divergence than unbiased genes. Bottom: Scaled divergence (dN/dS). Overall female‐biased genes evolve the fastest between species, followed by male‐biased, and finally unbiased genes. Groups with different letters are significantly different from each other, with values a > b > c, and colors follow the categorization from the methods.

Again, we first considered nonsynonymous substitutions per nonsynonymous site (dN). Again, sex‐biased gene classes evolved differently from each other ($X_{2}^{2}$ = 59.75, *p* < 0.00001). Once again, female‐biased genes held the most variation, but for dN, unbiased genes held intermediate, followed by male‐biased genes with the lowest divergence (Figure 3 top left, Table 4 Top). Then we considered synonymous substitutions per synonymous site (dS), which also varied significantly by sex‐bias class ($X_{2}^{2}$ = 153.85, *p* < 0.00001). The pattern here differs substantially from that in pS, with unbiased genes holding the most synonymous substitutions and male‐ and female‐biased genes holding significantly less (Figure 3 top right, Table 4 Middle). To explain this unexpected pattern, we characterized the proportion of genes showing zero synonymous substitutions: 17.7% of female‐biased genes had no synonymous substitutions, 8.1% of male‐biased genes, and only 4.1% of unbiased genes. And finally, we found strong differences in rates of divergence across the sex‐bias classes based on a Kruskal–Wallis test ($X_{2}^{2}$ = 509.11, *p* < 0.00001). For overall scaled divergence (dN/dS), female‐biased genes evolve the fastest, followed by male‐biased, then unbiased genes (Figure 3 bottom, Table 4 Bottom).

**TABLE 4:** Holm‐Bonferroni adjusted *p*‐values for pairwise differences between sex‐bias classes for between‐species variation (divergences).

Statistic	Female‐biased vs. unbiased	Unbiased vs. male‐biased	Female‐biased vs. male‐biased
dN	**< 0.0001**	**0.018**	**< 0.0001**
dS	**< 0.0001**	0.085	**0.004**
dN/dS	**< 0.0001**	**< 0.0001**	**< 0.0001**

*Note:* Bolded values are significant at a *p* < 0.05 threshold. Top row: Nonsynonymous substitutions per nonsynonymous site (dN). Middle: Synonymous substitutions per synonymous site (dS). Bottom: Scaled divergence (dN/dS).

### 3.5 Adaptation Across the Mealybug Genome

**FIGURE 4:**
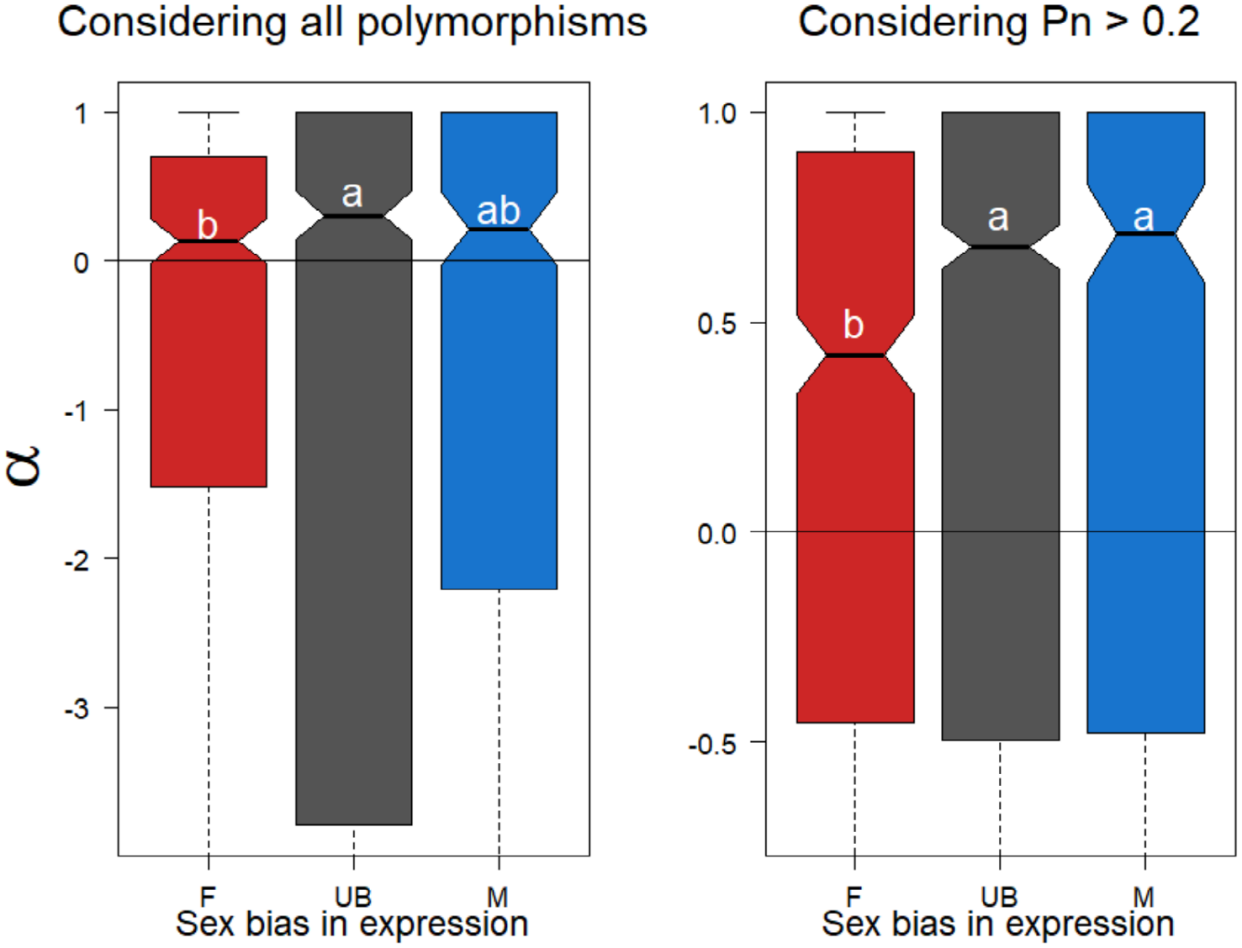
Adaptive evolution of sex‐biased genes under PGE. The proportion of substitutions driven by positive selection (*α*) when considering all polymorphisms (left) or excluding nonsynonymous polymorphisms with frequency < 0.2 (right). The latter should upwardly bias alpha if weakly deleterious variants segregate below this frequency. In both cases, female‐biased genes show less adaptation than unbiased genes, although male‐biased genes cannot be differentiated from either class when not filtering polymorphisms. After this filter, however, both male‐biased and unbiased genes evolve significantly more adaptively than female‐biased genes. We checked the robustness of our findings by further increasing the stringency to exclude nonsynonymous polymorphisms with frequency < 0.4 but found the same pattern of less adaptation in female‐ than unbiased or male‐biased genes (Figure S2).

We calculated *α* first in a simple way, utilizing all polymorphism data from our focal population, and found a significant difference between the sex‐bias classes ($X_{2}^{2}$ = 7.92, *p* = 0.019). Post hoc testing revealed that this result is driven by lower adaptation in female‐biased genes compared to unbiased genes (*p* = 0.016). Male‐biased genes did not evolve strongly differently than female‐biased genes (*p* = 0.093) or unbiased genes (*p* = 0.930, Figure 4 Left). We noted that *α* estimates were very low, with many per‐gene values being negative, a symptom of weakly deleterious polymorphisms sorting at low frequencies in the population (Charlesworth and Eyre‐Walker 2008). To account for this bias, we removed nonsynonymous polymorphisms below 0.2 frequency in our dataset and recalculated *α*. This approach upwardly biases the point estimate of *α*, but in a way that is proportional to the fraction of nonsynonymous polymorphisms segregating below the threshold value; in other words, the more low‐frequency nonsynonymous polymorphisms in a class of genes, the higher the adjusted *α* should become. With this approach, values more strongly differed ($X_{2}^{2}$ = 21.54, *p* = 0.0002). Here, female‐biased genes show less adaptation than either unbiased genes (*p* = 0.0.00001) or male‐biased genes (*p* = 0.0036). Male‐biased and unbiased genes do not evolve differently (*p* = 0.706, Figure 4 Right).

### 3.6 Gene Conservation Across Deeper Evolutionary Time

Of the 7332 consistently sex‐biased genes, we identified 2878 with a 1‐to‐1 ortholog with the Chinese wax scale, *E. pela*. When parsing these orthologs by sex bias in *P. citri* , we found a significant difference between the classes ($X_{2}^{2}$ = 842.94, *p* < 0.0001). In particular, nearly half (2542, 49.3%) of unbiased genes showed strict conservation, compared to 259 (25.8%) of male‐biased and only a mere 67 (5.7%) of female‐biased genes.

## 4 Discussion

### 4.1 New Genomic Resources for the Citrus Mealybug in Context With Other Mealybugs

We built upon the newly generated chromosomal assembly (Ross et al. 2024) to add value to the citrus mealybug as a model system. We generated 19 new RNAseq datasets, one proteomic dataset, and a set of gene annotations built on these new data. There are very few comparable sequencing efforts in mealybugs, but our annotations are consistent with recent resources generated for the obscure mealybug, *Pseudococcus viburni*, which has roughly 24,000 annotated protein‐coding genes (Vea et al. 2021) and stands in contrast to the annotation of the cotton mealybug, *Phenacoccus solenopsis*, with a mere 12,000 genes (Li et al. 2020). While the latter genome is roughly 30% smaller in total size than that of either *P. viburni* or *P. citri* , a near two‐fold difference in gene content is unlikely to be purely biological. And as both *P. citri* and *P. viburni* have BUSCO duplication rates < 10%, it is unlikely that the higher gene counts are substantially inflated by assembly artifacts. Instead, it is more likely that either the low number in *P. solenopsis* is an underestimate or the higher numbers reflect an increased rate of transposons annotated as genes in the species with larger genomes. Indeed, only around 15,000 genes were well‐represented in our RNAseq, which may be closer to the true number of protein‐coding genes in *P. citri* , but with so few mealybug genomes available, it is currently difficult to generalize patterns of genomic natural history at present.

### 4.2 Sexual Dimorphism, Paternal Genome Elimination, and Mealybugs

With these resources established, we turn to evolutionary genetic questions, starting with the question of how much differential gene expression there is between the sexes. As expected based on gross morphology, citrus mealybugs are also highly sexually dimorphic at the gene expression level. Roughly one‐third of consistently expressed genes were significantly sex‐biased and just over 60% were sex‐biased in at least one life stage (adults or nymphs). Within this latter class, the vast majority of partially sex‐biased genes show biased expression in adults but not juveniles. This observation fits well with predictions of sexual conflict theory. In general, the fitness interests of both sexes are expected to be aligned in immature individuals as both sexes need to survive and grow; these interests diverge more sharply at sexual maturity when sex‐specific reproductive strategies become relevant (Wedell et al. 2006). However, this simplified view overlooks the fact that to reach dimorphic adult phenotypes, initially similar juveniles must strongly diverge as they mature. In principle, this divergence in phenotype (and thus underlying gene expression) could extend earlier into development with more dimorphic species in order to accommodate large changes or could occur very sharply during development while minimizing the effects on juveniles.

Our expression data suggest the latter in the citrus mealybug, and indeed, these molecular observations match well with other unusual biology of the system. Despite being members of a hemimetabolous (i.e., gradually developing) order of insects, male mealybugs undergo a process similar to metamorphosis, known as neometaboly, which fundamentally and rapidly changes their body plan at the end of their nymphal stages with the addition of more developed sensory organs (e.g., eyes) and wings (Vea and Minakuchi 2021). Internally, adult males lose the endosymbiotic bacteria that provide juveniles of both sexes and adult females with essential nutrients (Kono et al. 2008). Neometaboly is common across mealybugs and the wider clade of scale insects (Vea et al. 2019), almost all of which are highly sexually dimorphic (Kosztarab and Watson 1994; Mongue et al. 2021, 2024), suggesting that this may be an ancestral developmental solution to generating wildly different adult phenotypes.

Returning to mealybugs, recent theoretical work has predicted PGE should create a more favorable evolutionary dynamic for the invasion of female‐biased alleles than male‐biased, which over time should lead to feminization of gene expression (Hitchcock et al. 2022). Lacking data for a closely related non‐PGE outgroup, we cannot confirm that such patterns are limited to PGE, but we can assess how well results in the citrus mealybug align with predictions for the system. And indeed, we see more female‐biased genes than male‐biased genes in *P. citri* . In terms of more familiar chromosomal sex determination, this places PGE's expression profile closer to X chromosome systems (Perry et al. 2014; Prince et al. 2010) and sets it apart from Z chromosome species, which are often observed to have more male‐biased than female‐biased gene expression (Mongue et al. 2022; Mongue and Baird 2024). More to the point, the identification of sex‐biased and unbiased genes allows us to answer the questions of molecular evolution that motivate this study.

### 4.3 Short‐Term Variation: Ploidy of Expression Creates Sex Differences

Both nonsynonymous and synonymous polymorphism individually followed the pattern of female‐biased genes holding the most variation, followed by male‐biased, then finally unbiased genes holding the least. Female‐biased genes represent the more familiar diploid baseline from Mendelian genetic systems, so the overall results are best viewed as reductions in variation in unbiased and male‐biased genes, both of which are exposed to haploid selection at least some of the time. This pattern matches observations that haploid expression in males strongly purges deleterious variants in other arthropods (Henter 2003; Tien et al. 2015) and is aligned with these expectations for PGE mealybugs (de la Filia et al. 2015). More surprisingly, this pattern extends to synonymous variants as well. As synonymous variants are rarely under direct selection (except, e.g., biased codon usage, Hershberg and Petrov 2008), this decrease in variation is likely the effect of background selection, which can remove neutral variants in linkage with sites under purifying selection (Charlesworth 2012). This possibility is supported by the observation that only one sex, females, has recombination in mealybugs, lowering the absolute rate of recombination compared to more familiar models (Bongiorni et al. 2004).

Parsing more finely, we saw even within haploid‐expressed genes that male‐biased genes hold more variation than unbiased genes. One traditional explanation for this pattern is that, being expressed in only half the population, male‐biased genes should experience relaxed selection compared to unbiased genes (Dapper and Wade 2016; Gershoni and Pietrokovski 2014). While this general logic holds true, for male‐biased genes under PGE, the reduced opportunity for selection should be even more strict; any given male‐biased gene currently expressed in a male will *not* be expressed in the next generation, needing to pass through a daughter in order to be expressed again in a grandson (de la Filia et al. 2015, 2021). As evidence for this reduced window of selection, we note that excess, likely deleterious polymorphisms reach higher frequencies (~0.3) in male‐biased genes than in either unbiased or female‐biased genes (for which variation drops off above ~0.1).

Finally, combining patterns of nonsynonymous and synonymous variants, we found that scaled polymorphism (pN/pS) was higher in female‐biased genes, but unbiased and male‐biased genes held similar ratios of variation. In summary, at the population level, patterns of variation based are consistent with differences in selection created by PGE based on the ploidy of expression. This result also sets an expectation for how long‐term variation should be distributed in the absence of positive selection (Kimura 1979; McDonald and Kreitman 1991; Ohta 1992).

### 4.4 Long‐Term Evolution: Sex Differences in Speed and Adaptation

We explored longer‐term evolutionary differences first through patterns of divergence, fixed differences between *P. citri* and *P. ficus* . We found that female‐biased genes had the highest scaled divergence (dN/dS), followed by male‐biased genes, with unbiased genes evolving the slowest. This overall dynamic belies differences between how nonsynonymous and synonymous substitutions accrue, however. First, on the nonsynonymous side, the haploid‐expressed genes evolve more slowly than female‐biased genes, likely because of increased selective scrutiny. Furthermore, any particular male‐biased allele can only be exposed to selection every other generation, which likely slows positive selection relative to unbiased genes, which can be expressed every generation. For synonymous divergence, the increase in substitutions in unbiased genes relative to female‐biased genes may be the result of hitchhiking of neutral variation pulled to fixation when in linkage with a beneficial variant under positive selection (Maynard‐Smith and Haigh 1974). This effect should be stronger in haploid‐expressed genes, where even recessive variation is under selection, but for male‐biased alleles, the requirement to pass through a daughter in between generations of selection likely gives more time for recombination to break up linkage between neutral and selected variants. Putting these patterns together, unbiased genes have the lowest dN/dS not because of a lack of nonsynonymous change, but rather because of a concomitant increase in synonymous divergence. Finally, it is worth noting that the patterns described above were found for sex‐biased genes, which do not necessarily have sex‐limited expression; however, to the extent that a sex‐biased gene is expressed in the opposite sex, this should decrease the difference in selection between the bias classes. In other words, we recovered strong differences despite some potential expression in both sexes. Evidence for the consistency of this pattern can be seen in our supplementary analysis of a small set of genes with truly sex‐limited expression, which show the same patterns described above.

More generally, the fact that relative rates of polymorphism and divergence differed between sex‐bias classes suggests differing selective forces acting on these gene classes (McDonald and Kreitman 1991). To directly examine adaptation, we estimated the proportion of adaptive substitutions, *α*, between *P. citri* and *P. ficus* for each class of genes. When considering all the polymorphisms in our dataset, we recovered fewer adaptive substitutions for female‐biased genes than unbiased genes, but with male‐biased genes indistinguishable from either group. When removing low‐frequency, likely deleterious, polymorphisms that violate the assumptions of the *α* statistic (Charlesworth and Eyre‐Walker 2008), we found that both unbiased and male‐biased genes evolve more adaptively than female‐biased genes. This difference could be observed even if the true proportion of adaptive substitutions between *Planococcus* species is the same across gene classes, but we more effectively excluded weakly deleterious variation from our calculations in male‐biased genes than female‐biased ones, thanks to their lower frequency in the former. Likewise, we found evidence for codon bias between male‐ and female‐biased genes. It has been suggested that codon bias might be related to the ploidy of expression (Singh et al. 2005), and thus our results may reflect more efficient selection on synonymous variants in male‐biased genes. In other words, despite the predicted (Hitchcock et al. 2022) and observed feminization of the genome (this study), we found greater evidence for selective scrutiny of genes expressed in *males*.

Combined with the above results on dN/dS, this suggests that male‐biased gene evolution is characterized by more adaptive, but slower change between species. To explore this dynamic over more distant evolutionary relationships, we compared conservation of 1‐to‐1 orthologs between *P. citri* and *Ericerus pela*, another PGE species from a separate hemipteran family, Coccidae (Yang et al. 2019). Using the sex‐bias classifications from *P. citri* , we found the most conservation of unbiased genes, followed by male‐biased genes, with female‐biased genes in *P. citri* showing the fewest 1‐to‐1 orthologs between species, consistent with the pattern seen in *Planococcus* species and suggesting that these dynamics are consistent across long evolutionary time under a common PGE sex determination system.

### 4.5 Comparison to Chromosomal Sex Determination

In traditional chromosomal sex determination systems, sex‐biased genes are often observed to evolve more adaptively on the sex chromosomes than autosomes (Kousathanas et al. 2014; Meisel and Connallon 2013; Mongue et al. 2022; Sackton et al. 2014), typically attributed to haploid selection on the X/Z in one sex. Conversely, when no increased adaptation is observed, the lower effective population size of the sex chromosome is often invoked to explain its absence (Mongue and Baird 2024). The PGE system is a useful contrast to the evolutionary dynamics of sex chromosomes for the simple reason that there is no tension between ploidy of expression and effective population size; these dynamics are consistent across the genome. Under these more straightforward conditions, we see patterns consistent with enhanced purifying selection on unbiased and male‐biased genes, as expected if haploid expression increases selective scrutiny of variants.

Less straightforward is the observation that some diploid‐expressed genes do show increased adaptation on sex chromosomes compared to autosomes (e.g., female‐biased X‐linked genes: Meisel and Connallon 2013; or male‐biased Z‐linked genes: Mongue et al. 2022). On the one hand, if these sex‐biased genes are still expressed in the heterogametic sex to some extent, haploid selection may still confer an adaptive benefit. To the extent that patterns of selection represent the primary sex of expression, however, there is no ploidy benefit to being sex‐linked in these cases. Instead, the simplest explanation is that, because of the biased transmission dynamics of sex chromosomes, sex‐biased genes are more likely to find themselves in the correct sex for a favorable selective background if they become linked to a sex chromosome. This logic underpins the prediction (Klein et al. 2021) and observation that the X should accumulate female‐biased genes and the Z should accumulate male‐biased genes (Mongue and Walters 2017). Indeed, because of the transmission dynamics of PGE, the whole genome should favor the invasion of female‐biased alleles (Hitchcock et al. 2022). As a driver of adaptation, however, our data suggest it is far less powerful than haploid selection, because female‐biased genes in *P. citri* show the lowest proportion of adaptive substitutions.

To truly confirm that these results are driven by ploidy of expression differences under PGE, and not idiosyncrasies of scale insects, will require study of other independently evolved PGE clades. At present, there are six independent origins known across arthropods (Herbette and Ross 2023; Ross et al. 2022). These clades are united by the common feature of elimination of paternal chromosomes but differ in ways that create natural experiments. For instance, the coffee berry borer beetle, *Hypothenemus hampei*, has mechanistically similar PGE, with paternal chromosomes heterochromatinized in males (Brun et al. 1995) but the sexes are phenotypically much less dimorphic than most scale insects (Perdana Harahap et al. 2020). Alternatively, predatory mites do not just silence the paternal genome but eliminate it entirely early in male development (Nelson‐Rees et al. 1980), making them fully haplodiploid. Future population genetic explorations of these and other PGE clades will be key to determining which molecular outcomes are common across this rare reproductive system.

### 4.6 Implications for Pest Management

Finally, mealybugs (Pseudococcidae) are the second largest family of scale insects (Hemiptera: Sternorrhyncha: Coccomorpha), with many species, including *P. citri* , being agricultural and ornamental pests of economic importance (Liebhold et al. 2024). A variety of control strategies have been developed, including chemical control (Franco et al. 2009) as well as more environmentally sustainable alternatives like mating disruption, mass‐trapping, attract‐and‐kill, augmentative biological control, and classic biological control (Beltra et al. 2015; Sullivan et al. 2019; Franco et al. 2022; Gilliéron et al. 2024). Some of these tactics target one sex in particular, such as mating disruption targeting adult males or the release of biological control parasitoids that seek female hosts. To be sure, more targeted studies of the evolutionary outcomes of mealybug pest management will be required, but our findings make testable predictions for the efficacy of different approaches. In particular, management that targets traits expressed in both sexes should be more likely to be effective across species, as the underlying genes appear more conserved over evolutionary time. Conversely, approaches that target only one sex, particularly females, may have more species‐limited effects because of the faster divergence of female‐biased genes. However, given the evidence for less adaptation in these same genes, mealybug pests may be less likely to evolve resistance to female‐targeted management practices. Integrating these molecular evolutionary considerations may help to develop robust control strategies with long‐term efficacy.

## 5 Conclusions

We have generated resources and undertaken population genetic analyses of the citrus mealybug, *P. citri* , to better understand how sex‐biased genes evolve under a non‐chromosomal sex determination system. We found that (1) sex‐biased genes, especially female‐biased genes, evolve faster than genes expressed in both sexes; (2) haploid expression in unbiased and male‐biased genes slows molecular change, likely from an increase in purifying selection; (3) in spite of the lower rate of change, the proportion of adaptive substitutions is higher for these unbiased and male‐biased genes and (4) unbiased genes in particular are well‐conserved across deep evolutionary time, with fewer male‐biased and especially female‐biased genes sharing a conserved ortholog with the soft scale, *E. pela*. Putting all of these observations together, our results suggest that PGE may slow evolution but not adaptation in males, causing female‐biased genes to vary more at multiple scales of evolutionary time. These results are consistent with theory developed in chromosomal sex determination systems, albeit without as many confounding factors that sometimes obscure them in those systems. Further study of non‐PGE scale insects and other independently evolved PGE clades will be required to confirm the causal role of the sex determination system as the main driver of these molecular evolutionary differences, but this study offers the first genome‐wide evidence in support of this hypothesis.

## Author Contributions

A.J.M. designed the experimental approach, performed genomic and population genetic analyses, and wrote the main and supplemental text. T.E.W. carried out gene expression analyses and wrote a portion of the main text. H.M. designed the gene expression experimental design, reared insects, and guided expression analyses. A.Gcarlos. and J.P.M. designed and carried out proteomic data collection and insect rearing. J.C.F. collected population genetic samples, contributed to sampling approach development, and wrote a portion of the main text. L.R. conceived of the overall project ideas, coordinated with co‐authors, and provided extensive feedback on the text. All authors reviewed and contributed to editing the text.

## Conflicts of Interest

The authors declare no conflicts of interest.

## Supporting information


Appendix S1.

## Data Availability

We have archived the data used for this project as follows. The genome assembly was previously published and can be accessed with the accession GCA_950023065.1 on NCBI. The gene annotation is hosted alongside analysis scripts at the following git repo: https://github.com/amongue/citriPopGen. Short‐read population genetic sequences are found under the NCBI BioProject PRJNA1211521 and RNA sequencing under PRJNA1207926.
