# Supplementary material for: Contrasting Evolutionary Trajectories Under Paternal Genome Elimination in Male and Female Citrus Mealybugs
Source: Mol Ecol. 2025 Jun 9;34(13):e17826. doi: 10.1111/mec.17826 (PMC12186724; doi:10.1111/mec.17826)
Supplement: Supplementary file 1 — Appendix S1. [file MEC-34-e17826-s001.docx]

**Supplementary analyses and results**

**Classification and study of genes with inconsistent sex-bias.**

In our examination of sex-bias across nymphs and adults, we identified a number of genes that differed in classification between stages. Here we consider these genes in more detail. Because we are more interested in the ploidy of expression and selection than particular life-history differences between juveniles and adults, we collapsed the off-diagonal categories with the same name for analysis. For example, in subsequent analyses, we combined genes that were nymph male-biased/adult unbiased with nymph unbiased/adult male-biased, as both sets of genes experience selection in both sexes in one stage and males only in another.

|  | Adult male-biased | Adult unbiased | Adult female-biased |
| --- | --- | --- | --- |
| Nymph male-biased | Male-biased | **Partially male-biased** | **Sex reversal** |
| Nymph unbiased | **Partially male-biased** | Unbiased | **Partially female-biased** |
| Nymph female-biased | **Sex reversal** | **Partially female-biased** | Female-biased |

**Table S1. Assignment of sex-biased genes based on expression profiles in both nymphal and adult mealybugs.** We completed differential expression analyses between the sexes for nymphs and adults separately, then compared results. Categories along the diagonal showed consistent expression patterns, while those off-diagonal disagreed between nymphs and adults. The latter categories were binned in subsequent analyses.


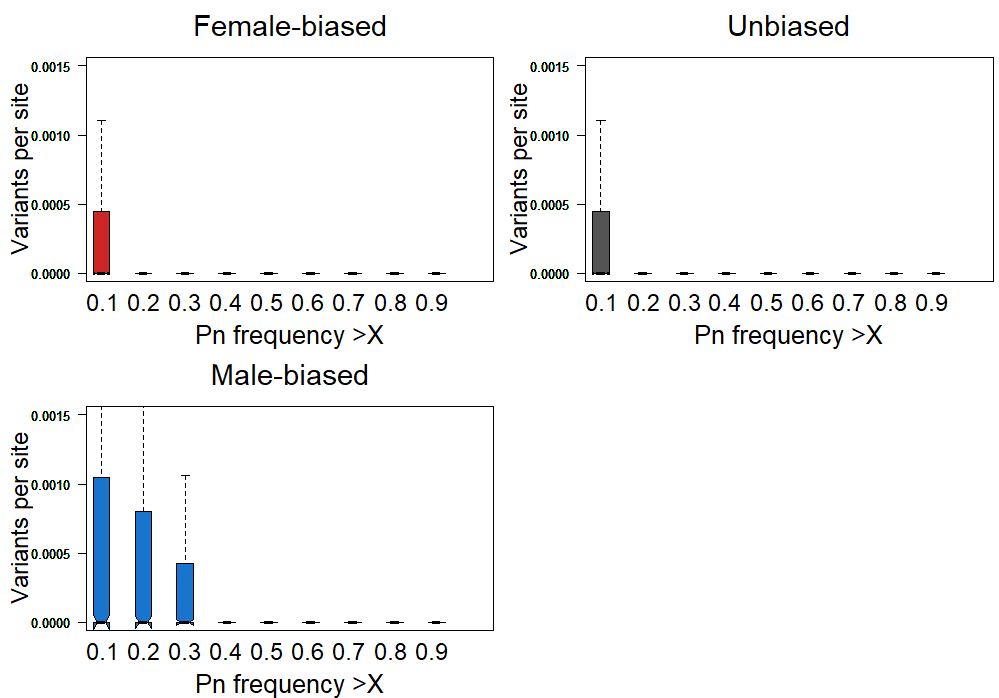


**Figure S1. Derived allele frequency of non-synonymous polymorphisms by sex-bias class.** All three gene classes show a massive excess of variation at low frequency (<0.2), suggesting a genome-wide phenomenon of weakly deleterious variation maintained in the population. For male-biased genes(bottom left) however, this excess of variation is maintained to much higher frequencies (<0.4), potentially due to the lack of direct transmission between fathers and sons.

| pN | Female-biased | Partially Female-biased | Unbiased | Partially Male-biased | Male-biased |
| --- | --- | --- | --- | --- | --- |
| Female-biased |  |  |  |  |  |
| Partially Female-biased | **<0.0001** |  |  |  |  |
| Unbiased | **<0.0001** | **<0.0001** |  |  |  |
| Partially Male-biased | **<0.0001** | **<0.001** | 0.871 |  |  |
| Male-biased | **<0.0001** | **0.046** | 0.678 | 0.490 |  |
| Bias reversal | 0.296 | 0.689 | **0.0007** | **0.0014** | **0.0440** |

**Table S2.** Holm-Bonferroni adjusted p-values for pairwise differences between sex bias classes for nonsynonymous variants per nonsynonymous site. Bolded values are significant at a p < 0.05 threshold.

| pN/pS | Female-biased | Partially Female-biased | Unbiased | Partially Male-biased | Male-biased |
| --- | --- | --- | --- | --- | --- |
| Female-biased |  |  |  |  |  |
| Partially Female-biased | **<0.0001** |  |  |  |  |
| Unbiased | **<0.0001** | **<0.0001** |  |  |  |
| Partially Male-biased | **<0.0001** | **<0.0001** | 0.500 |  |  |
| Male-biased | **<0.0001** | **0.0040** | 0.514 | 0.832 |  |
| Bias reversal | 0.486 | 0.483 | **<0.0001** | **<0.0001** | **0.0018** |

**Table S3.** Holm-Bonferroni adjusted p-values for pairwise differences between sex bias classes for scaled polymorphism (pN/pS). Bolded values are significant at a p < 0.05 threshold.


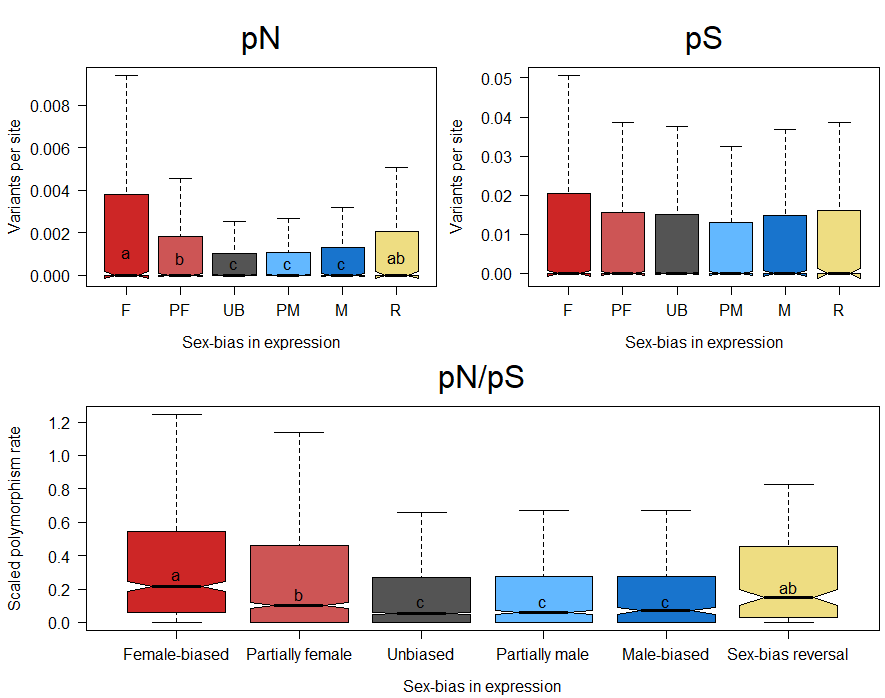


**Figure S2. Polymorphism and sex-biased expression across all sex-bias classes**. Colors correspond to the bias classes defined in Table S1 and letters denote significance such that groups sharing a letter are not statistically different from each other. **Top left:** Nonsynonymous variants per nonsynonymous site (pN). Female-biased genes hold the most variation, followed by partially female-biased genes, with bias reversed genes in between the two. Unbiased, partially and fully male-biased genes hold the least variation. **Top right:** synonymous variants per synonymous site (pS). There were no significant differences between any bias classes for synonymous variation. **Bottom:** Scaled polymorphism (pN/pS). With no differences in pS, results follow pN above, female-biased genes hold the greatest relative rate of nonsynonymous variation, with unbiased and male-biased genes holding the least.

We found only slight variations in inferred α (the proportion of adaptive substitutions). None of these was significantly different from the rest based on overlap of 95% confidence intervals (Table X).

| α | α point estimate | 95% CI | Significant differences |
| --- | --- | --- | --- |
| Female-biased | 0.072 | [0.005, 0.154] | NA |
| Partially female | 0.117 | [0.062, 0.168] | NA |
| Unbiased | 0.127 | [0.084, 0.169] | NA |
| Partially male | 0.072 | [0.008, 0.139] | NA |
| Male-biased | 0.072 | [-0.028, 0.161] | NA |
| Sex-bias reversal | 0.066 | [-0.078, 0.192] | NA |


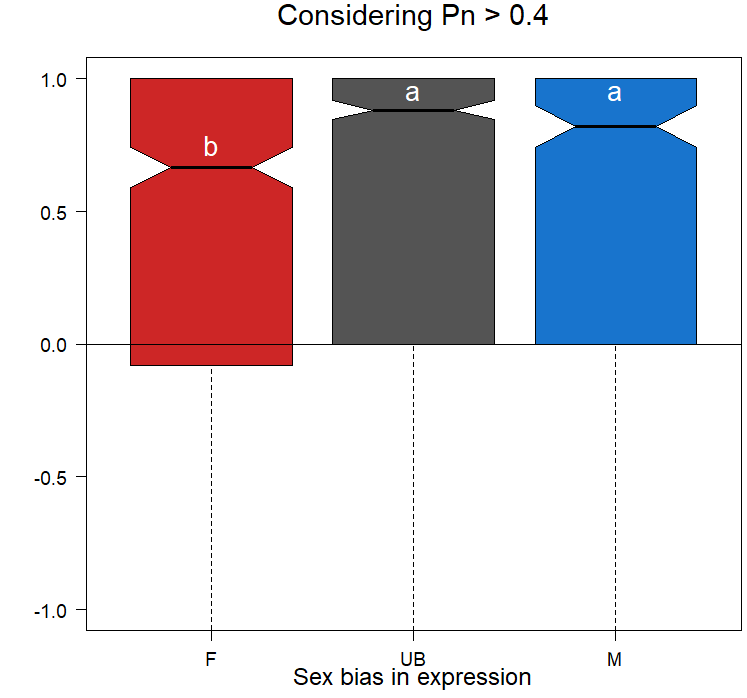


**Figure S3.**

*Special mention of sex-bias reversed genes*

Our investigation of both nymphal and adult mealybugs allowed us to categorize the consistency of sex-bias across life stages. The majority of genes, roughly 58% in our dataset, had the same expression class in both stages. For the rest of the expressed genes, most were unbiased in juveniles but showed sex-biased expression in adults and a smaller subset was sex-biased in juveniles but not adults. Finally, the smallest category, 413 genes total, was male-biased at one stage and female-biased in another. Studies of sex-biased genes across multiple observations, often between closely related species, often find a similar small set of genes that reverse sex-bias between groups (Ranz et al. 2003; Ellegren and Parsch 2007). As a proportion of the total, this category is small enough to be within the expectation of the Type I error rate, i.e., false positives, but is a large enough number of genes in our case that we explored them further.

In terms of molecular evolution, they showed more variance than other classes, often statistically indistinguishable from multiple other classes, unlike consistently male- or female-biased genes. In both the short and long term, bias-reversed genes fell in between strictly and partially female-biased genes but held more nonsynonymous variation than unbiased or (partially) male-biased genes, suggesting that the selective forces acting on them were more consistent with expression in females than males. Without clear biological reasons to expect this bias reversal or strong functional annotations to explore further, we end our investigation of these genes here and devote the bulk of our discussion to parsing patterns of more traditionally studied sex-bias classifications.

**Functional analysis of genes by bias class**

To further explore differences between the sexes, we ran a functional annotation of predicted genes using Interproscan v5.53-87.0 and intersected this information with the sex-bias categories defined in the main manuscript. Below we present the top 5 most enriched GO terms for each of the sex bias classes relative to the others. We also include the full list of annotations as a supplementary file (<https://github.com/amongue/citriPopGen> in the interproscan_results.zip).

| Female-biased | Unbiased | Male-biased |
| --- | --- | --- |
| GO:0007155  Cell adhesion | GO:0008270, GO:0005634  Zinc ion binding, nucleus | GO:0008017, GO:0005874, GO:0000226  Microtubule binding, microtubule, microtubule cytoskeleton organization |
| GO:0016491, GO:0031177, GO:0009058, GO:0016740, GO:0016746, GO:0006633, GO:0004315  Oxidoreductase activity, phosphopantetheine binding, biosynthetic process, transferase activity, acyltransferase activity, fatty acid biosynthesis process, 3-oxoacyl-[acyl-carrier-protein] synthase activity | GO:0016020, GO:0005509, GO:0007156, GO:0005886, GO:0007155  Membrane, calcium ion binding, homophilic cell adhesion via plasma membrane adhesion molecules, plasma membrane, cell adhesion | GO:0005515, GO:0005543  Protein binding, phospholipid binding |
| GO:0050660, GO:0016614, GO:0005509  Flavin adenine dinucleotide binding, oxidoreductase activity acting on CH-OH group of donors, calcium ion binding | GO:0003677  DNA Binding | GO:0005515, GO:0005886, GO:0007411, GO:0007155, GO:0007399, GO:0005887  Protein binding, plasma membrane, axon guidance, cell adhesion, nervous system development |
| GO:0005515, GO:0006979, GO:0020037, GO:0004601  Protein binding, response to oxidative stress, heme binding, peroxidase activity | GO:0005840, GO:0003735, GO:0006412  Ribosome, structural constituent of ribosome, translation | GO:0005515, GO:0007417, GO:0007155  Protein binding, central nervous system development, cell adhesion |
| GO:0016021, GO:0005509, GO:0016079  GO:0046907  Calcium ion binding, synaptic vesicle exocytosis, intracellular transport | GO:0005515, GO:0005509  Protein binding, calcium ion binding | GO:0005515, GO:0070588, GO:0016020, GO:0005262, GO:0055085, GO:0006811, GO:0005216  Protein binding, calcium ion membrane transport, membrane, calcium channel activity, transport membrane, monoatomic ion transport, monoatomic ion channel activity |

**Exploring sex-biased vs. sex-limited expression**

In the main manuscript, we define gene classes by significant differential expression between males and females in both nymphs and adults. This analysis gives us a robustly supported set of genes to analyze but does not tell us about the *degree* of sex-bias. For instance, both genes with 4:1 expression in males:females and 4:0 could be classified as male-biased. The former should mainly be exposed to selection in males while the latter is exclusively under selection in males. It is an open question whether these two distinct types of genes evolve differently. In other words, do sex-specific genes evolve differently than merely sex-biased genes?

To answer this question, we calculated the specificity metric (SPM) as the proportion of expression of each gene in females using the same RNAseq data as in the main manuscript and applied it to our subset of 7,322 genes with consistent bias. From this analysis, we subsetted only the genes with the strongest SPM bias (tails of SPM in Figure S4), then further selected only those genes for which our DEseq bias categories agreed; this created a small subset of genes for which we have the best evidence of consistent sex limited expression: 142 female-specific and 83 male-specific genes.


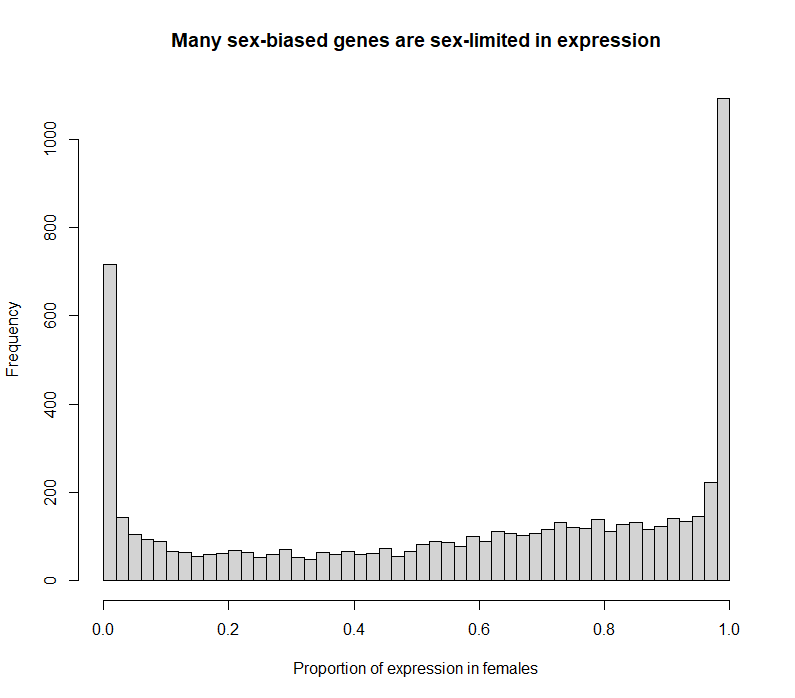


**Figure S4. Sex-specificity of genes used in main analyses.** Gene expression is highly dimorphic, with many sex-biased genes showing female-limited (SPM = 1) or male-limited (SPM = 0) expression.

Below, we explore the molecular evolution of these genes exclusively. As expected based on our overall sex-biased gene analyses, genes with female-limited expression hold more polymorphic variation (W = 2564, p = 0.044, Figure S5, left) and diverge faster than those with male-limited expression (W = 6846, p < 0.0001, Figure S5, right); however, with so few genes, we did not recover a significant difference in simple α (W = 1369, p = 0.297, Figure S6, top left). Even when removing non-synonymous polymorphisms of increasingly higher frequencies, we did not recover a significant difference between female- and male-limited genes, but the pattern of α values matched that found in the main text: excluding low-frequency variants has more of an inflating effect on male-limited genes than female-limited (Figure S6). Given the consistent pattern, the lack of significant difference in α is likely due to small sample sizes and high variation in individual gene α values.

To summarize, the patterns found short- and long-term variation in this small subset of genes with sex-specific expression are consistent with our broader observations from sex-biased genes.


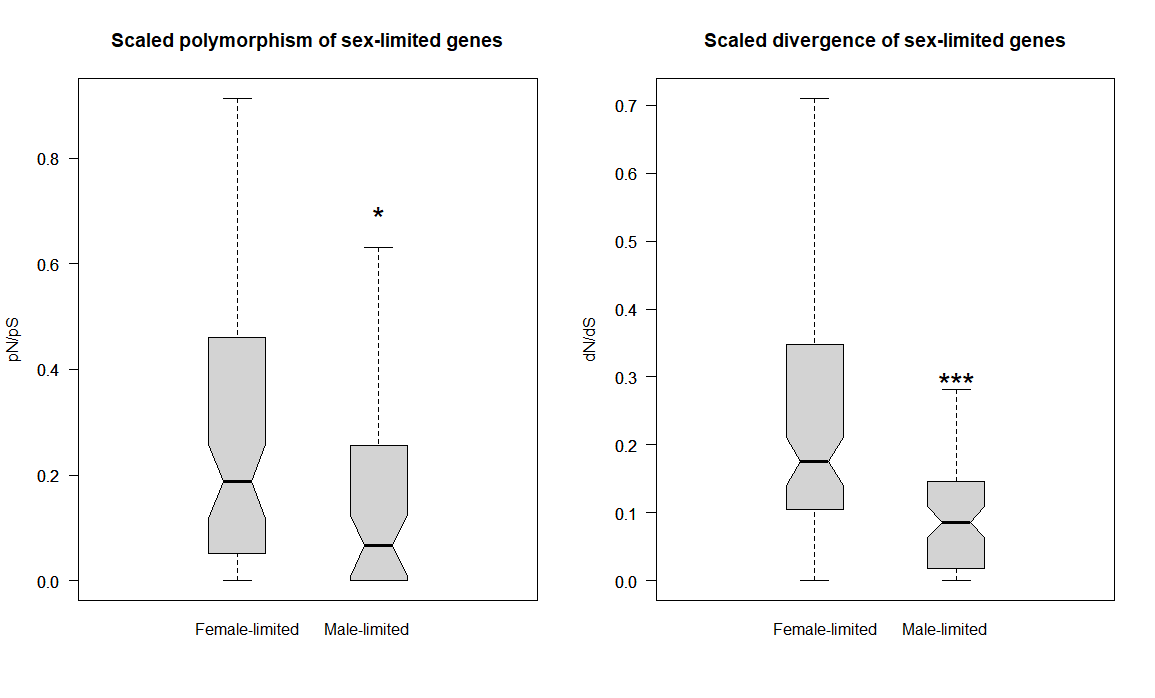


**Figure S5. Variation genes with sex-limited expression.** Male-limited genes hold less variation both within the population (left) and between species (right).


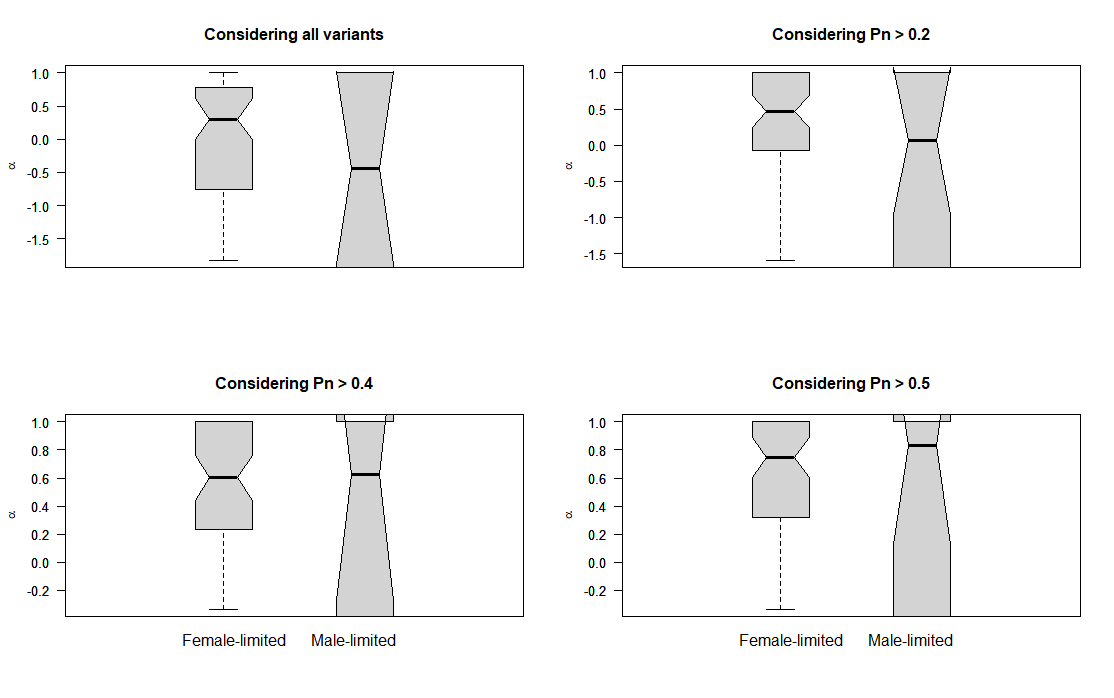


**Figure S6. Α values for genes with sex-limited expression, using different variant filtering thresholds.** In each plot female-limited genes are to the left of male-limited genes. None of the pairwise comparisons are significantly different from each other, but male-limited genes respond more to low-frequency variant filtering than female-limited genes do.

**Exploring codon bias**

To explore potential biased codon usage based on ploidy of expression, we examined the composition of basepairs for sites that were both third position and four-fold degenerate in our gene annotations, as defined by the previously mentioned degeneracy annotation scripts. For simplicity, we compared only male-biased genes, which should be predominantly haploid expressed, and female-biased genes, which should be predominantly diploid expressed. We tested for a significant difference in base composition between these two classes and found a strong difference (X^2^_3_ = 342.44, p < 0.0001, below).

| Bias class | A | C | G | T |
| --- | --- | --- | --- | --- |
| Female-biased | 31.57% | 19.32% | 18.18% | 30.93% |
| Male-biased | 29.59% | 19.59% | 20.12% | 30.70% |
